# Evolution of Cu and Zn speciation in agricultural soil amended by digested sludge over time and repeated crop growth

**DOI:** 10.1007/s11356-024-34784-8

**Published:** 2024-08-31

**Authors:** Jianting Feng, Ian T. Burke, Xiaohui Chen, Douglas I. Stewart

**Affiliations:** 1 School of Civil Engineering, University of Leeds, Leeds, LS2 9JT UK; 2 School of Earth and Environment, University of Leeds, Leeds, LS2 9JT UK

**Keywords:** Anaerobic digestor sludge, Agricultural soil, Evolution of metal speciation, Sequential extractions, X-ray absorption spectroscopy, Crop uptake

## Abstract

Metals such as Zn and Cu present in sewage sludge applied to agricultural land can accumulate in soils and potentially mobilise into crops. Sequential extractions and X-ray absorption spectroscopy results are presented that show the speciation changes of Cu and Zn sorbed to anaerobic digestion sludge after mixing with soils over three consecutive 6-week cropping cycles, with and without spring barley (*Hordeum vulgare*). Cu and Zn in digested sewage sludge are primarily in metal sulphide phases formed during anaerobic digestion. When Cu and Zn spiked sludge was mixed with the soil, about 40% of Cu(I)-S phases and all Zn(II)-S phases in the amended sludge were converted to other phases (mainly Cu(I)-O and outer sphere Zn(II)-O phases). Further transformations occurred over time, and with crop growth. After 18 weeks of crop growth, about 60% of Cu added as Cu(I)-S phases was converted to other phases, with an increase in organo-Cu(II) phases. As a result, Cu and Zn extractability in the sludge-amended soil decreased with time and crop growth. Over 18 weeks, the proportions of Cu and Zn in the exchangeable fraction decreased from 36% and 70%, respectively, in freshly amended soil, to 28% and 59% without crop growth, and to 24% and 53% with crop growth. Overall, while sewage sludge application to land will probably increase the overall metal concentrations, metal bioavailability tends to reduce over time. Therefore, safety assessments for sludge application in agriculture should be based on both metal concentrations present and their specific binding strength within the amended soil.

**Supplementary Information:**

The online version contains supplementary material available at 10.1007/s11356-024-34784-8.

## Introduction

Sewage sludge is the by-product of wastewater treatment (Lin et al. 2021). It contains valuable nutrients and organic matter (typically > 50% by dry weight) and is increasingly being applied to agricultural land as a fertiliser (Gomes et al. 2019; Rutgersson et al. 2020; Sun et al. 2022). Unfortunately, it also contains modest levels of heavy metals (Wang et al. 2021; Zhou et al. 2020). Typical metal concentrations in UK sludges are 1 mg/kg Cd, 46 mg/kg Cr, 176 mg/kg Cu, 19 mg/kg Ni, 77 mg/kg Pb and 432 mg/kg Zn (European Commision 2018). Similar concentrations are also found in sludges from other EU countries. In low- and middle-income countries, metal concentrations can be 4–5 times higher (Saha et al. 2018; Yang et al. 2014). As metal concentrations in sludges are usually higher than in agricultural soils, long-term repeated sludge application will inevitably lead to the gradual accumulation of heavy metals in soils (Achkir et al. 2023; Feng et al. 2023). Eventually, it may cause environmental and health risks (Chu et al. 2018; Gu and Wong 2004). This has led to the careful monitoring of total metal concentrations in agricultural soils to ensure they remain below prescribed limits (DEFRA 2018; EEC 1986). However, this control will restrict the agricultural use of sludges at a time when there is a pressing need to increase agricultural sustainability and reduce the dependence on mineral fertilisers. These regulatory limits are also increasingly being viewed as too simplistic as they do not recognise that the risk posed by metals in a particular soil is strongly dependent on their speciation (Adele et al. 2018; Feng et al. 2023). While total metal concentrations are a useful proxy for the risk, they provide no information on what percentage of sludge-introduced metals are bioavailable for crops, and so may potentially be taken up in foodstuffs (Ndzana et al. 2021). Thus, to better manage the agricultural use of sludges and control the risk, there is a need for information on the speciation and fate of metals associated with the sludge applied to the soil.

Generally, Zn and Cu are the two most abundant heavy metals in sewage sludge, so these metals will accumulate most quickly in the soil (You et al. 2020). Excessive Zn and Cu accumulation will adversely affect the soil quality and crop growth (Yen et al. 2024). Previous investigations indicate that Cu is principally bound to organic matter, and Zn is bound to carbonates, Fe–Mn oxides and organic matter and tightly bound to minerals in sewage sludge (Illeraa et al. 2000; Morera et al. 2001), whereas Cu and Zn are both primarily tightly bound to the minerals in agricultural soil (Doelsch et al. 2006; Jin et al. 2017). In sludge-soil mixtures, Cu and Zn binding is typically intermediate between these two components (Feng et al. 2023).

Additionally, long-term field studies have shown that sewage sludge has land productivity benefits, such as increased nutrient availability, but it also increases heavy metal concentrations in the soil, and importantly, it can increase the proportion of heavy metals that are bioavailable relative to untreated control soils (Kidd et al. 2007). The long-term field studies after cessation of sludge application have also shown that crop uptake of Zn and Cd in a given season is proportional to the soil concentrations, and that a proportion of the Zn and Cd in the soil remains bioavailable over more than 20 years (Mcgrath and Cegarra 1992; McGrath et al. 2000). More recent studies investigating metal speciation in the sludge-soil mixtures have typically been conducted over relatively short periods following sludge application, generally a few days (Malinowska 2017; Parvin et al. 2022; Wu et al. 2006). Their aim was to understand the immediate change in metal speciation in the soil due to sludge use. It should be noted that when sewage sludge is applied to land, the metal speciation within the mixture will evolve over time due to organic matter degradation by soil microorganism, interaction with organic chemicals exuded by crops and the effects of microbial metabolism on host phases such as sulphides and iron oxides (Caracciolo and Terenzi 2021; De Conti et al. 2018; Gan et al. 2020). As a result, metals in the mixture may be transformed into more bioavailable forms or sequestered in unreactive minerals. Currently, there is a gap in knowledge between studies that show the immediate changes in heavy metal bioavailability when sludge is applied to soil and the very long-term studies that highlight the residual bioavailability of such metals.

Based on the above context, the primary objective of this study is to determine the evolution of Cu and Zn speciation in an agricultural soil amended by sludge application, over time, and with repeated growth of spring barley (*Hordeum vulgare*), using a combination of sequential chemical extractions and X-ray absorption spectroscopy (XAS). The accumulation patterns of Zn and Cu in crop roots and shoots were also identified. This work addresses the knowledge gap about the evolution of metal speciation during repeated crop growth and will inform the risk assessments for sewage sludge use in agriculture.

## Materials and methods

### Collection and preparation of materials

#### Collection of soil and sewage sludge

Sewage sludge was collected from Esholt Wastewater Treatment Works, a large municipal wastewater treatment plant in West Yorkshire, UK, serving ~ 760,000 people (Edina 2024). It was a secondary treatment sludge that had undergone further thermal hydrolysis (165 °C at 6 bar for 30 min) prior to anaerobic digestion. The soil was collected from a working arable field at Spen Farm, Tadcaster, UK (lat. 53.8699, long. − 1.3290) on 24 August 2021 when the field contained a mature crop of maize (taken at 8 randomly selected locations between the crops). The field has been annually ploughed and cropped with conventional management since 1995 (Berdeni et al. 2021). The typical fertiliser inputs to the field are detailed in Holden et al. (2019). The soil is a Cambisol, with a silt loam texture containing stone fragments derived from the underlying dolomitic limestone (Guest et al. 2022; Humphries et al. 2023). The soil and sludge were stored at 4 ℃ prior to the experiments. Sub-samples of soil and sludge were air-dried, disaggregated by Retsch RS200 Disc Mill and sieved by a 106-µm sieve for characterisation prior to any experiments.

#### Preparation of amended sludge

To produce a sludge-amended soil where changes in Cu and Zn speciation could be easily determined by both sequential extractions and XAS, the original sludge was spiked with ZnCl_2_ and CuCl_2_. Preliminary investigations (see Tables S1–S3 and Fig. S1 in SI section S1) determined that linear sorption behaviour was largely maintained up to 2 wt% of Cu and Zn. Therefore, a ~ 20,000 mg/kg Cu and Zn amended sludge was produced for use in soil amendment experiments. This process ensured the vast majority of Cu and Zn present in the sludge-soil experiments were absorbed into the sludge prior to mixing or crop growth.

#### Preparation of sludge-amended soil

The sludge spiked with ~ 20,000 mg/kg of both Zn and Cu was added to the original soil at a typical ratio of 5 wt%. The sludge and soil were mixed for ~ 5 h in a Hobart mixer (model A200) and then allowed to stand for 21 days. This produced sludge-amended soil containing ~ 1000 mg/kg Zn and Cu, where ~ 90% of the Cu and Zn were introduced with the amended sludge.

### Germination of spring barley seeds

Spring barley seeds (supplied by Cotswold Seeds Ltd) were germinated between two filter papers soaked with half-strength Murashige and Skoog nutrient solution (2.2 g/L, pH = 7.0) on petri dishes (5 seeds per dish). The petri dishes were sealed with parafilm and kept in the dark at room temperature for 2 days to allow the seeds to germinate.

### Pot trial

Plug-trays containing square prismatic “pots” were used (top, 5 cm × 5 cm; bottom, 3.5 cm × 3.5 cm; height, 4.5 cm). Each pot was equipped with two layers of needle-punched polypropylene geotextile (non-woven; supplied by Spudulica) at the bottom to avoid soil loss from drainage holes. Each pot was filled with 95 g amended soil (initial moisture content, 15.3%). The crop seedlings were transplanted to the pots by placing them in a prepared hole (0.5–1 cm deep), and then the loose surrounding soil was used to cover the roots (1 seedling per pot in the first growth round). The planted pots (48 pots) and controls (12 pots) were placed in a laboratory growth chamber for 6 weeks. The growth chamber consisted of a steel frame covered with lighttight Mylar foil and equipped with 3 strips of LED growth lamps that delivered 18.3–25.6 µmol/m^2^/s of photosynthetically active radiation (400–700 nm range) at the level of the soil surface. The lamps were on a timer with a 12-h on/off cycle. During the growth phase, the soil moisture content in the plug-trays was maintained at 35% (about 70–80% of the field capacity) by regular irrigation with distilled water.

After the completion of the first round (6 weeks), crops were harvested. The soils recovered from the planted pots (bulk soil) and controls (control soil) were collected separately. Bulk soil and control soil from all replicates were combined and mixed separately. Sub-samples of each soil were taken after re-homogenisation for subsequent analysis. The remaining bulk and control soils were used separately for crops and controls in the second and third rounds. The second and third rounds followed the same procedures, except two seedlings were planted in each planted pot (38 planted pots and 10 controls in the second round; 32 planted pots and 8 controls in the third round).

### Sampling

#### Crops

The crop height (from soil surface to the tip of the crop flag leaf) was recorded every 7 days during the growth period. After 6 weeks, the crops were harvested, and the crop root system was carefully removed from the planted pots. All the crop roots were then put into a 1-l beaker containing 600–700 ml distilled water, which was placed in an ultrasonic bath for 30 min to dislodge any soil particles from the roots. The washing procedure was repeated 10 times. Crop shoots were also washed with distilled water. The washed crops were oven-dried at 105 ℃ for 30 min and then at 60 ℃ for a further 72 h. The dry weight biomass of roots and shoots was measured separately. Then the shoots and roots were ground in a Retsch CryoMill and stored at 4 ℃ for future analysis.

#### Sludges and soils

The entire volume of soil suspension obtained from the sonification of the roots in each growth round was evaporated in an oven at 60 ℃. Any root fragments in this root-bound soil were removed, and the remaining root-bound soil was prepared for aqua regia digestion and European Community Bureau of Reference (BCR) sequential extractions (the root-bound soil was not retained from the first round).

Samples of original soil, original sludge, bulk soil and control soil from the three rounds were each divided into two parts. One part was prepared for BCR and aqua regia digestion. The other part was freeze-dried, ground to a fine powder and prepared as 8-mm pressed pellets held in Kapton™ tape for XAS analysis. The amended sludge and the amended soil (before pot trial) were each divided into three parts. Two parts were treated as described above. The last part was dried by solvent displacement and then embedded in 30-mm polished epoxy resin blocks to allow scanning electron microscopy-energy dispersive x-ray spectroscopy (SEM–EDS) and microfocus X-ray fluorescence (µXRF) analysis.

### Analytical methods

#### Physico-chemical analysis

pH was measured by the 1:2.5 solid/water (*w*/*v*) suspension method using a calibrated pH metre (Jordan-Vidal et al. 2020). The organic matter content was measured by the loss on ignition (Gerenfes et al. 2022). Total Kjeldahl nitrogen and total phosphorus content were determined using the total nitrogen kit (Hach APC338) and total phosphorous kit (Hach APC350), respectively, using a Hach AP3900 laboratory robot (Al-Haddad 2022).

#### Aqua regia digestion analysis

Total metal concentrations in soils, sludges and crops were determined by aqua regia digestion on triplicate samples (Turek et al. 2019). For soil and sewage sludge, ~ 0.2 g of sample was weighed into a conical flask and 10 ml aqua regia was added. The flask was heated on a hot plate to achieve effervescence and then for a further 30 min. After cooling, the contents of the conical flask were transferred to a 100-ml volumetric flask. Distilled water was added to a volume of 100 ml. This suspension was filtered (0.45-μm syringe filter), and the solution was analysed by VARIAN 240 FS Atomic Absorption Spectrophotometer (AAS; optimum working ranges, 0.03–10 µg/ml for Cu at a wavelength of 324.7 nm and 0.01–2 µg/ml for Zn at a wavelength of 213.9 nm). For roots and shoots (which effervesce excessively in aqua regia), ~ 0.2 g was first digested in hydrochloric acid (5 ml) at room temperature and then evaporated to a dry residue. On cooling, this residue underwent aqua regia digestion, as described above.

To ensure comparability with other laboratories, total Zn and Cu concentrations in a certified reference material (LSKD-2, typical lake sediments from various locations within the Canadian Shield, provided by CCRMP, CANMET Mining and Mineral Sciences Laboratories) were determined by this aqua regia digestion procedure (full details see Table S4 in SI section S2). In addition, the precision of this aqua regia digestion procedure for Cu and Zn has been demonstrated to be < 7%, with an accuracy of 95–105%, meeting the criteria for satisfactory precision (≤ 20%) and accuracy (80–120%) in aqua regia digestion (Chen and Ma 2001).

#### BCR extraction analysis

BCR sequential extractions used in this study were developed from the procedures of Yang et al. (2018). The extractions were conducted on 0.5 g of dry soil/sludge in 50-ml polypropylene centrifuge tubes. Specific steps are shown in Table 1. BCR analyses were also conducted on triplicate samples, and the mass balance on the recovery of metals was calculated (the amounts of metals in the four fractions divided by the amount obtained by total digestion in aqua regia).

**Table 1:** BCR sequential extractions adopted for the determination of metal speciation

Step	Fraction	Extractant	Shaking time	Temperature
F1	Exchangeable	20 ml of 0.11 M CH_3_COOH (pH = 5)	16 h	Room temperature
F2	Reducible	20 ml of 0.5 M NH_2_OH·HCl (pH = 2)	16 h	Room temperature
F3	Oxidizable	5 ml of 30% H_2_O_2_ (pH = 2)	1 h	Room temperature
		/	1 h	85 ± 2 ℃
		A further aliquot of 5 ml of 30% H_2_O_2_ (pH = 2)	1 h	85 ± 2 ℃
		25 ml of 1.0 M CH_3_COONH_4_ (pH = 2.0)	16 h	Room temperature
F4^a^	Residual	10 ml of aqua regia	0.5 h (after effervescence)	Heating on a hot plate

^a^The residue from F3 was analysed using the same procedure as used for the total metal concentrations

#### SEM–EDS analysis

Selected sludge/soil samples were mounted in epoxy resin blocks, before being ground and polished with diamond pastes using water-free oil-based lubricant. Before SEM analysis, the samples were coated with 15–20 nm carbon by thermal evaporation. SEM–EDS was performed on a Tescan Vega3 XM SEM at Leeds Electron Microscopy and Spectroscopy Centre, University of Leeds (beam energy 20 keV, working distance 15 mm). This SEM is equipped with an Oxford Instruments X-max 150 SDD EDS using Aztec software. The elemental mapping was performed at a spot size resolution of 2 µm. Based on the elemental maps, some interest spots were selected for further analysis.

#### XAS and µXRF analysis

Cu and Zn K-edge (8979 and 9659 eV, respectively) X-ray absorption near edge structure spectra (XANES) and multielement µXRF maps were collected on beamline I18 at the Diamond Light Source, UK (see SI section S3). For each sample, multiple scans were averaged to improve the signal-to-noise ratio using Athena version 0.9.26 (Ravel and Newville 2005). All XANES spectra were normalised and corrected to account for drift in *E*_0_ using Cu- and Zn-metal reference spectra. Linear combination fitting (LCF) using selected standards spectra (information of all reference standards shown in Fig. S2, Table S5 and S6) was performed to estimate the proportional contribution of different atomic coordination environments present in sample spectra. Standards used for Cu LCF analysis were Cu(II)CO_3_, Cu(II)SO_4_, Cu(II)O, Cu(I)_2_O, covellite, Cu(II)-humic complex, Cu^2+^ (aqueous), Cu(II)(CH_3_COO)_2_, Cu(I)S nanoparticles, Cu(II)_3_(PO_4_)_2_, Cu(II)(OH)_2_ and Cu(II)-hydrous ferric oxide. Standards used for Zn LCF analysis were Zn(II)SO_4_, Zn(II)O, sphalerite, Zn(II)-FeOOH, Zn(II)S nanoparticles, Zn(II)CO_3_, Zn(II)(CH_3_COO)_2_, Zn(II)_3_(PO_4_)_2_, Zn^2+^ (aqueous), Zn(II)-humic complex, Zn(II)-hydrous ferric oxide and Zn(II)(OH)_2_.

#### Statistical analysis

The statistical analysis was performed by one-way analysis of variance (ANOVA) at a 5% significance level to compare the mean values of experimental data using SPSS version 26.0. Students’ *t*-test was used to determine the significant level wherever required.

## Results

### Characterisation of original sewage sludge and agricultural soil

The average pH values for original sewage sludge and agricultural soil were 7.9 and 7.5, respectively (see Table S7). The organic matter content of sewage sludge was 51.1% whereas that of the soil was 6.6%. The total Kjeldahl nitrogen and total phosphorus concentrations in sewage sludge were about 75,500 mg/kg and 30,200 mg/kg whereas their soil concentrations were about 2900 mg/kg and 700 mg/kg, respectively. Total Cu and Zn concentrations in sewage sludge were about 303 mg/kg and 620 mg/kg and those in the soil were about 78 mg/kg and 114 mg/kg. The metal concentrations in the soil and sludge are both within the regulated limits of the UK (regulations of agricultural soil; the use of sewage sludge is limited by the maximum allowable metal concentrations in soil) and US guidelines (regulations on the use of sewage sludge in agriculture), respectively (DEFRA 2018; EPA 2022).

### Crop analysis

#### Crop height

**Fig. 1:**
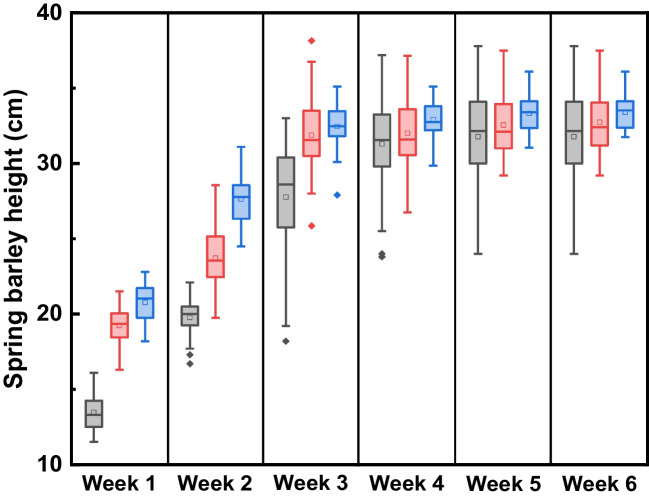
The recorded crop height during growth period (grey, first round; red, second round; blue, third round; shaded boxes show the median values and interquartile range; tails indicate 1.5 × IQR; square (□) indicates mean value; shaded diamond (♦) indicates outliers)

Similar patterns of spring barley (*Hordeum vulgare*) growth were observed in the three growth rounds (see Fig. 1). Specifically, significant growth occurred in the first 3 weeks (*P* < 0.05), followed by only minor growth in the fourth and fifth week. The height remained largely unchanged in the sixth week. After 6 weeks, the average height of crops for the three growth rounds was 31.8 cm, 32.7 cm and 33.4 cm, respectively.

#### Dry weight biomass

The dry weight biomass of crop shoots was more than two-fold greater than that of the roots in each growth round (see Table 2). When there were two crops per pot, the ratio of shoot-to-root biomass was greater than that of a single crop in a pot. The dry weight biomass per crop of three growth rounds was 0.13 g/plant, 0.16 g/plant and 0.17 g/plant, respectively.

**Table 2:** Dry weight biomass of crop roots and shoots

Growth round	Dry weight biomass of shoots (g)	Dry weight biomass of roots (g)	Survived planted pots number	Harvested crops number	Dry weight biomass of each crop shoot (g/plant)	Dry weight biomass of each crop root (g/plant)
1st round	3.8	1.8	40	40	0.09	0.04
2nd round	8.7	2.6	37	74	0.12	0.04
3rd round	7.9	3.1	32	64	0.12	0.05

#### Zn and Cu uptake by crops

**Fig. 2:**
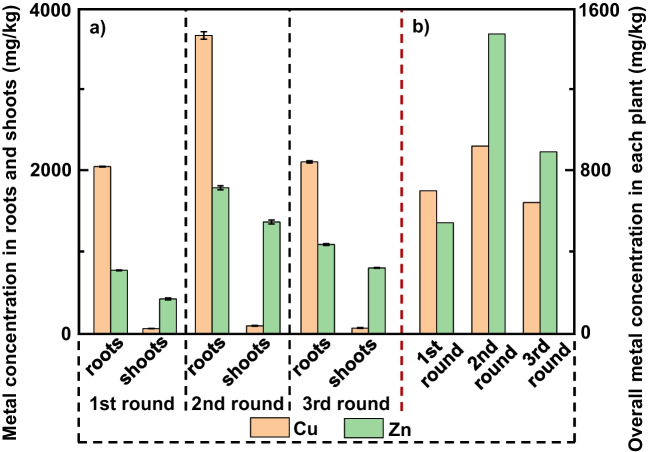
**(a)** Zn and Cu concentrations in crop roots and shoots of three growth rounds and (**b**) overall Zn and Cu concentrations in each plant

The average Cu and Zn concentrations in whole crops across the three growth rounds were about 760 and 970 mg/kg (see Fig. 2). The Cu concentration in whole crops during the first growth round was slightly higher than Zn. However, in the second and third rounds, the Zn concentration in whole crops was higher than Cu. In total, < 1% Cu and Zn in the soil was removed over three growth rounds. The ratio of the Cu concentrations in the roots and shoots was 30 ± 4 (mean ± standard deviation) which indicates that crop-associated Cu was strongly partitioned towards the roots. In contrast, the root-to-shoot ratio for Zn concentrations was 1.5 ± 0.3 (mean ± standard deviation). When corrected for the amount of dry biomass, the overall Zn uptake to shoots was approximately 60% of the total biomass associated Zn, whereas overall Cu uptake to shoots was only about 8% of the total biomass associated Cu.

### Zn and Cu speciation in sludges and soils

#### Cu and Zn concentrations in soils

The Cu and Zn concentrations in the control soil and the bulk soil determined by aqua regia digestion were within ± 3% of their concentrations in the amended soil in all three growth rounds, as shown in Tables 3 and 4 (ANOVA indicated no statistically significant difference at *P* < 0.05). The total Cu concentration in the root-bound soil was ~ 3% lower than in the bulk soil in the second growth round (a *t*-test indicated that this difference was significant at *P* < 0.05) and ~ 7% lower in the third growth round (likewise, this difference was significant). The total Zn concentration in the root-bound soil was ~ 6% higher than in the bulk soil in the second growth round (statistically significant at *P* < 0.05) but was ~ 2% lower in the third growth round (not statistically significant at *P* < 0.05).

**Table 3:** Cu concentration of each fraction in different samples (mg/kg of dry soil/sludge)

Samples	Cu concentration in each fraction (mg/kg)				Sum of four fractions (mg/kg)	Total metal content by aqua regia (mg/kg)	Recovery rate (%)
	CH_3_COOH extract	NH_2_OH·HCl extract	H_2_O_2_ extract	Aqua regia extract			
Original soil	11 ± 1	9 ± 1	13 ± 2	42 ± 2	74 ± 4	78 ± 5	96
Amended sludge	2724 ± 52	2398 ± 72	11,699 ± 377	2690 ± 464	19,511 ± 173	18,520 ± 202	105
Amended soil	380 ± 4	356 ± 4	270 ± 16	61 ± 4	1067 ± 12	1058 ± 23	101
Control_1st round	340 ± 4	356 ± 11	291 ± 9	66 ± 3	1053 ± 16	1035 ± 7	102
Control_2nd round	318 ± 14	359 ± 14	301 ± 8	65 ± 1	1043 ± 24	1057 ± 5	99
Control_3rd round	298 ± 1	423 ± 6	275 ± 7	73 ± 2	1070 ± 14	1067 ± 23	100
Bulk_1st round	303 ± 15	423 ± 6	272 ± 9	68 ± 1	1067 ± 10	1066 ± 34	100
Bulk_2nd round	264 ± 7	442 ± 11	279 ± 8	70 ± 2	1055 ± 21	1068 ± 6	99
Bulk_3rd round	255 ± 5	413 ± 7	299 ± 10	80 ± 2	1047 ± 10	1064 ± 19	98
Root soil_2nd round	240 ± 3	386 ± 10	314 ± 11	76 ± 3	1016 ± 2	1032 ± 5	98
Root soil_3rd round	240 ± 5	375 ± 4	296 ± 2	88 ± 5	999 ± 2	993 ± 3	101

All values are expressed as mean ± standard deviation (*n* = 3)

**Table 4:** Zn concentration of each fraction in different samples (mg/kg of dry soil/sludge)

Samples	The concentration of Zn in each fraction (mg/kg)				Sum of four fractions (mg/kg)	Total metal content by aqua regia (mg/kg)	Recovery rate (%)
	CH_3_COOH extract	NH_2_OH·HCl extract	H_2_O_2_ extract	Aqua regia extract			
Original soil	1 ± 1	4 ± 2	2 ± 0	105 ± 3	112 ± 1	114 ± 5	99
Amended sludge	14,291 ± 272	2676 ± 159	354 ± 71	366 ± 71	17,687 ± 476	17,694 ± 129	100
Amended soil	720 ± 9	193 ± 7	28 ± 5	90 ± 4	1031 ± 3	1065 ± 24	97
Control_1st round	683 ± 4	219 ± 2	47 ± 0	92 ± 2	1041 ± 8	1034 ± 9	101
Control_2nd round	655 ± 10	239 ± 5	51 ± 2	95 ± 0	1040 ± 15	1070 ± 9	97
Control_3rd round	602 ± 5	278 ± 7	46 ± 1	97 ± 3	1022 ± 17	1048 ± 12	98
Bulk_1st round	629 ± 21	281 ± 17	39 ± 1	106 ± 7	1056 ± 10	1050 ± 42	101
Bulk_2nd round	565 ± 12	322 ± 13	46 ± 2	107 ± 8	1039 ± 16	1080 ± 16	96
Bulk_3rd round	526 ± 6	292 ± 7	68 ± 2	106 ± 3	992 ± 9	1042 ± 15	95
Root soil_2nd round	498 ± 13	370 ± 2	102 ± 7	117 ± 10	1087 ± 9	1144 ± 13	95
Root soil_3rd round	481 ± 5	317 ± 3	89 ± 1	108 ± 0	995 ± 7	1026 ± 2	97

All values are expressed as mean ± standard deviation (*n* = 3)

#### Cu and Zn distribution in amended sludge and amended soil

**Fig. 3:**
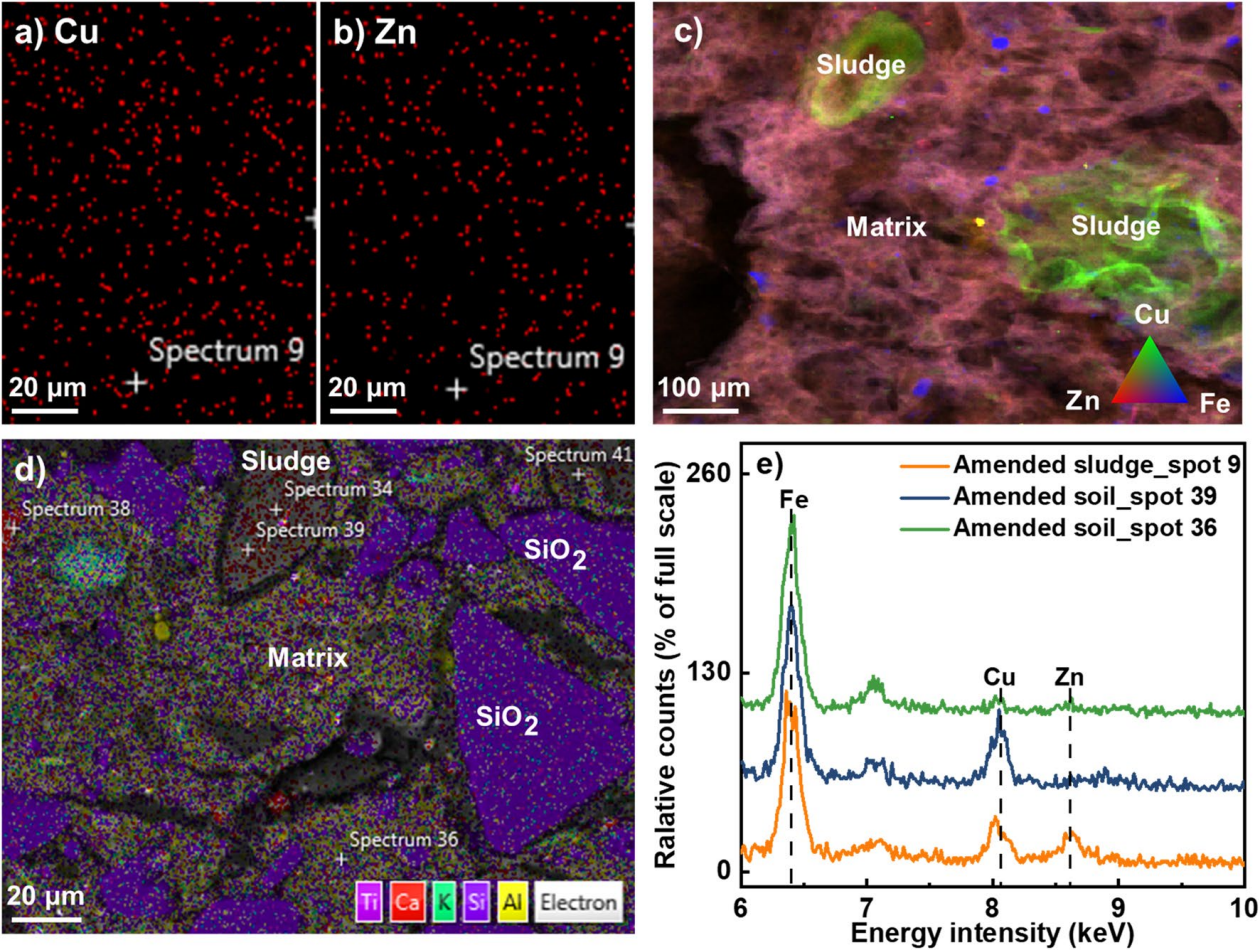
SEM–EDS elemental maps showing uniform distribution of both (**a)** Cu and (**b)** Zn in the amended sludge (from same area of interest); (**c)** false colour µXRF map showing the distribution of Cu, Zn and Fe in the amended soil and (**d)** false colour SEM–EDS map of the amended soil (different areas of interest); (**e)** truncated EDS spectra collected from the locations indicated in maps (**a)**, (**b)** and (**d)**

SEM–EDS elemental maps showed Cu and Zn were uniformly distributed in the amended sludge (see Fig. 3). When the amended sludge was added to the soil, remnant sludge particles were observed in the amended soil 21 days after mixing that had preferentially retained Cu relative to Zn. Similar features were seen in the µXRF maps of the amended soil. The µXRF maps further showed that Zn (and the remainder of the Cu) were evenly distributed within the soil matrix (which had an elemental composition consistent with a mix of Fe oxides and clay minerals).

#### Cu speciation changes

**Fig. 4:**
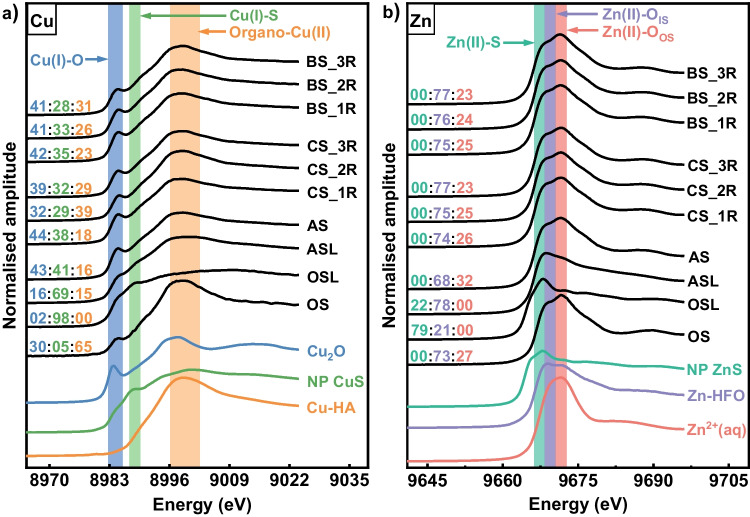
Bulk average K-edge XANES spectra for (**a)** Cu and (**b)** Zn collected from different samples and selected standards. Ratios presented above sample spectra are results for LCF analysis of bulk sample spectra. Colours match to the standards shown. BS/CS_1R/2R/3R, bulk/control soil from the first/second/third round; AS, amended soil; ASL, amended sludge; OSL, original sludge; OS, original soil. NP, nanoparticles; Cu-HA, Cu-humic acid complex; Zn^2+^ (aq), aqueous Zn^2+^; Zn-HFO, Zn-hydrous ferric oxide; O_IS_ and O_OS_, inner sphere and outer sphere binding to metal oxide surfaces

LCF of Cu XANES spectra (Fig. 4a and Table S8) indicated that Cu(I)-O (30%), Cu(I)-S (5%) and organo-Cu(II) (65%) atomic binding environments predominated in the original soil, whereas in the original sludge, Cu(I)-S containing phases were dominant (98%). 69% of the Cu in the amended sludge was also in a Cu(I)-S binding environment, but 16% was in Cu(I)-O and 15% was in organo-Cu(II) binding environments. In the amended soil 21 days after mixing, there were roughly equal amounts of Cu(I)-O (43%) and Cu(I)-S (41%) containing phases, with the remaining 16% present as organo-Cu(II) phases.

In the control soil, the proportion of Cu in Cu(I)-O and Cu(I)-S phases decreased over 18 weeks to 39% and 32%, respectively, and the proportion of Cu in organo-Cu(II) phases increased to 29%. A similar change in Cu speciation was seen over the same period with repeated crop growth. The proportion of Cu in Cu(I)-O phases and Cu(I)-S phases decreased to 41% and 28%, respectively, and the proportion of Cu in organo-Cu(II) phases increased to 31%.

**Fig. 5:**
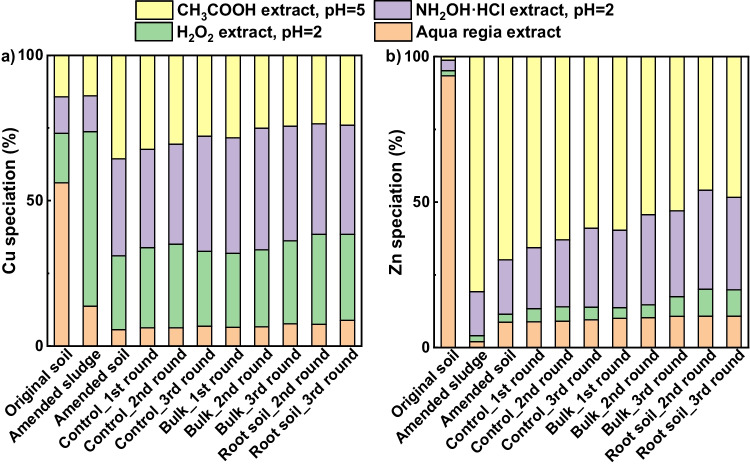
Evolution of operationally defined (**a)** Cu and (**b)** Zn speciation over time and with repeated crop growth

BCR analysis showed that 56% of the Cu in the original soil was in the aqua regia extraction (see Fig. 5a), with smaller proportions distributed between the CH_3_COOH (14%), NH_2_OH·HCl (13%) and H_2_O_2_ extractions (17%). In contrast, 78% of the Cu in the original sludge was in the H_2_O_2_ extraction, with most of the remainder (16%) in the aqua regia extraction (see Fig. S1). The distribution of Cu in the amended sludge was similar to that in the original sludge, with 60% in the H_2_O_2_ extraction and 10–15% in each of the other extractions. In the amended soil, 36% of the Cu was in the CH_3_COOH extraction, 33% was in the NH_2_OH·HCl extraction and 25% was in the H_2_O_2_ extraction, with only 6% in the aqua regia extraction.

In the control soil, the proportion of Cu in the CH_3_COOH extraction decreased significantly over 18 weeks to 28% (*P* < 0.05). The proportion of Cu in the NH_2_OH·HCl extraction increased significantly to 40% while the proportions in the H_2_O_2_ and aqua regia extractions were 26% and 7% after 18 weeks. A similar change in Cu distribution occurred with repeated crop growth in the bulk soil. However, the proportion of Cu in the CH_3_COOH extraction decreased significantly more than in the control soil to 24% (*P* < 0.05). The proportion of Cu in the NH_2_OH·HCl extraction increased by a similar amount to the control soil to 39% (which is not statistically different from the control soil). The proportion in the H_2_O_2_ extraction increased significantly more than in the control soil to 29%.

As there is a net loss of Cu from the root-bound soil, the proportions in each extraction from the root-bound soil have been calculated relative to the amount of extractable Cu in the amended soil. In both rounds, the proportion of Cu in the CH_3_COOH extraction was 24%, while 38% was in the NH_2_OH·HCl extraction, nearly 30% was in the H_2_O_2_ extraction and 5–10% was in the aqua regia digestion.

#### Zn speciation changes

LCF of Zn XANES spectra (Fig. 4b and Table S9) indicated that the Zn was in one of two distinct Zn(II)-O binding environments in the original soil. 27% of the Zn was in a binding environment similar to the hydrated aqueous Zn^2+^ solution species, which is also present in outer sphere binding complexes (Zn(II)-O_OS_), while 73% was in a binding environment similar to inner sphere binding to metal oxide surfaces (Zn(II)-O_IS_). In contrast, Zn(II)-S phases predominated (79%) in the original sludge, with the remaining Zn in a Zn(II)-O_IS_ binding environment (21%). The same two Zn(II) binding environments were present in the amended sludge as in the original sludge, but the proportions were reversed (78% Zn(II)-O_IS_ and 22% Zn(II)-S). The Zn binding environments in the amended soil were similar to the original soil (32% Zn(II)-O_OS_ and 68% Zn(II)-O_IS_), and the Zn(II)-S binding was absent.

In the control soil, there was a significant reduction in the proportion of Zn(II)-O_OS_ phases to 26% after 6 weeks, with only a small further decrease in the remaining two experimental rounds. Concurrently, the proportion of Zn(II)-O_IS_ phases increased to 74% after one round, with a small further decrease in the subsequent rounds. The same changes in the Zn-binding environment were also seen in the bulk soil.

BCR data showed that 93% of the Zn in the original soil was in the aqua regia extraction (Fig. 5b). In contrast, 37% of the Zn in the original sludge was in the H_2_O_2_ extraction, with 31%, 20% and 12% in the NH_2_OH·HCl, aqua regia and CH_3_COOH extractions, respectively (see Fig. S1), whereas in the amended sludge Zn was primarily in the CH_3_COOH extraction (81%), with most of the remainder in the NH_2_OH·HCl extraction (15%). The distribution of the Zn in the amended soil was similar to the distribution in the amended sludge, with 70% in the CH_3_COOH extraction and 19% in the NH_2_OH·HCl extraction, 3% in the H_2_O_2_ extraction and 9% in the aqua regia extraction.

In the control soil, the proportion of Zn in the CH_3_COOH extraction decreased significantly over 18 weeks to 59%, while the proportion of Zn in the NH_2_OH·HCl extraction increased significantly to 27%, with little change in the other extractions (*P* < 0.05). A similar pattern of change in Zn distribution occurred with repeated crop growth in the bulk soil. However, the proportion of Zn in the CH_3_COOH extraction decreased by significantly more than in the control soil to 53% (*P* < 0.05), while the proportion in the NH_2_OH·HCl extraction increased to 29% (which is not significantly different from the control soil). There were also small but statistically significant increases in the proportion of Zn in the other two extractions.

As it is inappropriate to assume that there is no Zn flux to/from the root-bound soil, the proportions of Zn in each extraction from the root-bound soil have been calculated relative to the total amount of extractable Zn in the amended soil. In the root-bound soil, the proportion of Zn in the CH_3_COOH extractions was between 45 and 50%, while the proportion in the NH_2_OH·HCl extractions was between 35 and 30%, with ~ 10% of the Zn in each of the other two extractions.

## Discussion

### Speciation of Cu and Zn in anaerobic digestor sludge and agricultural soils

Sequential extractions on the digested sludge showed that Cu was predominately in the H_2_O_2_-extracted fraction (oxidizable). Similar operationally defined speciation has been reported for a wide range of sludges from several regions (Chen et al. 2008; Dabrowska 2016; Tytla 2020). This is generally attributed to the high affinity of Cu for organic matter and the high stability of Cu-organic matter complexes (Feng et al. 2023), although this has not been verified for digested sludge. However, XANES analysis indicates that Cu in digested sludge is primarily in Cu(I)-S phases, with no contribution from organo-Cu(II) complexes. This does not conflict with the BCR results as metal sulphides and metal–organic matter complexes are not easily separated in sequential extractions, with both commonly reporting to “oxidizable fraction” (Rodgers et al. 2015). Further, the formation of metal sulphides during anaerobic digestion has been postulated by some researchers (Dabrowska and Rosinska 2012; Feng et al. 2023; Thanh et al. 2016). Sequential extractions indicated that Zn in the digested sludge was fairly evenly split between all four operationally defined fractions, but XANES analysis indicates most Zn was in the Zn(II)-S binding environment, and the remainder in an environment characteristic of Zn(II) in an inner sphere complex with metal oxides. This probably reflects the inherent weakness of sequential extractions, where a metal phase can be extracted in different fractions depending on the degree of crystallinity (Rennert 2019).

The Cu binding environment in the amended sludge was similar to that in the original sludge with the majority in the Cu(I)-S environment, which is compatible with most of the Cu being extracted in the “oxidizable” fraction. However, Zn binding environments differed between the amended sludge and the original sludge, with the majority in inner-sphere complexes with metal oxides, and a small proportion in a Zn(II)-S binding environment, the reverse of the pattern in the original sludge. This may have arisen because Cu out-competed Zn for reactive sulphides in the amended sludge. This difference explains why a lower proportion of Zn in the amended sludge is extracted in the “oxidizable” fraction, but the increase in the “exchangeable” fraction (CH_3_COOH-extracted fraction). This in turn suggests that at least part of the Zn present in recently formed inner-sphere complexes on metal oxide surfaces can be extracted with the “exchangeable” fraction.

Cu and Zn were predominately organo-Cu(II) and Zn(II)-O_IS_ phases in the original soil. These major phases would be expected to be extracted by H_2_O_2_ (oxidizable fraction) and NH_2_OH·HCl extractants (reducible fraction), respectively (Yang et al. 2018). However, both metals reported predominantly to the “residual” fraction (aqua regia–extracted fraction; as found in other sequential extraction studies) (Kotoky et al. 2015; Topcuoğlu 2014). The low extractability of these Cu and Zn adsorption complexes from the original soil may be associated with “ageing” in situ, as presumably, they accumulated slowly over long timescales in agricultural soil. Clearly, soil-associated Cu and Zn are not readily mobilised to solution and are most likely to have low bioavailability, so as to pose a much lower environmental risk compared with these metals in sewage sludge.

### Processes occurring during the mixing of amended sludge and soil and over time

Remnant sludge particles were found in the amended soil 21 days after mixing by SEM–EDS mapping. The EDS spectrum further showed Cu concentrations in these remnant sludge particles were higher than Zn concentrations, indicating Zn is preferentially lost from the sludge over a relatively short period. In addition, Zn binding environments in the amended soil reflected a transition to “soil-like” speciation, with Zn still predominantly in Zn(II)-O_IS_ phases, but with complete loss of Zn(II)-S phases. In contrast, sequential extractions indicated a very small change in Zn extractability beyond what would be anticipated simply from a mixture of soil and the amended sludge. In comparison, Cu speciation in the amended soil reflected only a partial transition from “sludge-like” Cu binding environment towards the “soil-like” binding environment 21 days after mixing (possibly because Cu was retained more in the remnant sludge particles). There were increases in the proportions of organo-Cu(II) complexes and Cu(I)-O phases and a decrease in the proportion of Cu(I)-S phases that are larger than what would be anticipated from mixing. As would be anticipated from the addition of sludge, sequential extractions showed Cu mobility was higher in the amended soil than in the original soil, with the “exchangeable” and “reducible” Cu fractions increased, and the “oxidizable” and “residual” Cu fractions decreased.

There were small further changes in the Zn binding environment with time shown by the control experiments, although there was a steady decrease in Zn extractability with time. The proportion of Zn in the “exchangeable fraction” decreased, and there were very small increases in the other three fractions (most notably in the “reducible fraction”). The Cu binding environment also evolved slowly over time towards a “soil-like” state, with an increase in the proportion of organo-Cu(II) complexes and a decrease in the proportion of Cu(I)-S phases. Interestingly, after the initial increase in the proportion of Cu(I) associated with metal oxides upon mixing, that proportion decreased with more time towards that in the original soil, suggesting that this may be a transition speciation. Like Zn, sequential extractions also indicated a steady decrease in Cu extractability with the reduction in the proportion in the “exchangeable fraction” largely balanced by an increase in the “reducible fraction”. There was little change in the proportion in the H_2_O_2_ fraction probably because both organo-Cu(II) and Cu(I)-S are “oxidizable” extracted by this reagent. Therefore, over time, Cu and Zn speciation in the control soil slowly evolved, tending towards the metal-phase fractional distribution pattern seen in the original soil. This evolution process is probably attributable to the reactions of the metals with the more stable components in the soil and their slow incorporation into these components (Han and Banin 1999).

### Evolution of metal speciation with crop growth and metal uptake to crops

The XANES data from the bulk soil showed the changes in both Cu- and Zn-binding environments were similar with and without crop growth. However, the decreases in the extractability of both these metals were more rapid when crops were present (the concentration of both metals in the “exchangeable fraction” decreased). It is notable that the decrease in the extractability of both these metals was greater in the root-bound soil, where there were increases in the concentrations of Cu in the “residual fraction” and Zn in the “oxidizable fraction”.

The decrease in Cu and Zn in the “exchangeable fraction” of the control soil was principally the result of the transfer to the “reducible fractions” because there was no discernible reduction in the total Cu and Zn in the soil. The slightly larger decrease in Cu and Zn in the “exchangeable fraction” of the bulk soil resulted in more transfer of Zn but less transfer of Cu to the “reducible fraction”. There were also small transfers of both metals to the “oxidizable” and “residual” fractions. The root-bound soil exhibited the largest decrease in Cu and Zn in the “exchangeable fraction”, which was associated with a net decrease in the soil’s metal concentrations. This resulted in the most transfer of Zn but the least transfer of Cu to the “reducible fraction”, and slightly more transfer to the “oxidizable” and “residual” fractions. The more pronounced decrease in Cu and Zn in the “exchangeable” fraction of the root-bound soil is compatible with plant uptake being predominantly from the readily extractable phases (De Conti et al. 2018).

The finding that Cu and Zn concentrations were both higher in the roots than in the shoots has also been reported by Yu et al. (2011). With Cu, the average ratio of root-to-shoot concentration was very high (~ 30), whereas it was far lower for Zn (~ 1.5), indicating that Cu is strongly retained in the roots, but Zn is readily translocated into shoots. This tendency for Cu to be retained in crop roots has also been reported for other monocot and eudicot crop species by Hilber et al. (2007) and Juknys et al. (2009). In contrast, despite the total uptake of Cu and Zn being similar, less Zn is retained in the roots. It is probably because Zn is needed for the production of the growth hormone auxin (Deveshwar et al. 2020; Mapodzeke et al. 2021), which is mainly produced in crop shoot meristems (without this, hormone crop growth is compromised). In addition, the recommended dietary allowance for adults is tenfold higher for Zn than Cu (NIH 2022b), and the tolerable upper intake level is three- to fivefold higher for Zn than Cu (EFSA 2006; Government of Canada 2024; NIH 2022a). This means that elevated concentrations of extractable Cu and Zn in soil may pose a similar risk to food crops where the stem, leaves, flowers and fruit are harvested, but elevated concentrations of extractable Cu are potentially very problematic for root vegetables.

### Implications for anaerobic digestor sludge application to agricultural land

Anaerobic digestion is the most common method for treating sewage sludge before use in agriculture (SEL 2023). For example, ~ 70% of UK sewage sludge is treated by anaerobic digestion, and over 80% of this treated sludge is disposed to agricultural land (BAS 2024; Liu 2019). During anaerobic digestion, metal sulphides can become the primary host phase for chalcophile metals (seen in the Cu and Zn data presented above). Rapidly precipitated, poorly crystalline, metal sulphide phases are relatively reactive (Fu et al. 2014; Jeong et al. 2010; Wu et al. 2020), easily oxidised and more bioavailable than the agricultural soil-associated metals. Thus, the metals added with anaerobically digested sludge are likely to be rapidly transformed and redistributed to the soil matrix, but remain moderately mobilizable (at least for a time). Cu and Zn speciation evolved towards that seen in the original agricultural soil and this was slightly accelerated by repeated crop growth and by close association with crop roots, but the transformation rate was element-specific. With current data, it is difficult to quantify the transformation rate, and thus not possible to predict how long specific metals will remain mobile.

Currently, agricultural utilisation of sewage sludge is regulated by the total metal concentrations in the soil (DEFRA 2018; EEC 1986; Hudcová et al. 2019; MEE 1985). While these regulations are sufficient to prevent a large accumulation of metals from the sludge, they may be unduly conservative because metals in immobile fractions will not cause significant risk (Feng et al. 2023). In practice, “regular monitoring” of metals in the mobile fractions would better define the overall risk from sludge application to soils. Therefore, there is a need to update the current regulations taking into account bioavailability criteria for heavy metals. Limits could then be based on total leachable metals present during cropping rather than on bulk concentrations.

Additionally, the primary objective of this study was to determine how the speciation of Cu and Zn introduced to agricultural soil by sewage sludge evolves over time and with crop growth. To ensure that the changes in speciation determined by sequential extractions and XAS were principally due to changes in the speciation of the sludge-associated metals, the sludge used in this study was spiked with Cu and Zn to final concentrations about 50–100 times higher than the typical metal concentrations in UK sewage sludge (although within the range seen, historically, in heavily contaminated sludge) (Babel and del Mundo 2006; Jain and Tyagi 1992; Olthof and Lancy 1979). BCR and XAS data showed that Cu speciation in the amended sludge was broadly similar to that in the original digested sludge. Cu(I)-S was the dominant phase in both materials, although some Cu(I)-O and organo-Cu(II) phases were detected in the amended sludge that were not present in the original sludge. BCR extractions showed that most of the Cu was extractable by H_2_O_2_ in both the original and amended sludges, although a slightly higher proportion of Cu in the amended sludge was in the more easily leached fractions (which is compatible with the small differences in speciation determined by XAS). There were bigger differences in Zn speciation between the amended sludge and the original digested sludge. Nearly 80% of the Zn was in Zn(II)-S phases in the original sludge, with the remainder in a Zn(II)-O_IS_ binding environment. The same two Zn(II) binding environments were present in the amended sludge, but the proportions were reversed. BCR data showed that about 80% of the Zn in the amended sludge was in the exchangeable fraction, whereas it was distributed in the exchangeable, reducible, oxidizable and residual fractions in the original sewage sludge. Thus, the experiments reported in this study are likely to reflect the fate of Cu introduced to soil with sewage sludge reasonably well, whereas the results for Zn require more careful interpretation. However, the conversion of Zn(II)-S in the amended sludge predominantly to Zn(II)-O_OS_ upon mixing with the soil and the subsequent slow conversion of Zn(II)-O_OS_ to Zn(II)-O_IS_ over time reflects a transition in Zn speciation towards that seen in the original soil are likely to occur in a real system.

## Conclusion

Both Cu and Zn are present in digested sewage sludge primarily as metal sulphide phases formed during anaerobic digestion. These phases are relatively reactive, easily remobilised and more bioavailable than the soil-associated metals. Metals added to soils with the amended sludge are rapidly transformed and redistributed to the soil matrix but remain more easily mobilised (at least for a time). XAS showed that about 40% of Cu added as Cu(I)-S phases and all Zn added as Zn(II)-S phases in the amended sludge were converted to other phases after addition to the soil (mainly Cu(I)-O and outer sphere Zn(II)-O phases). Over time, both Cu and Zn are transformed from easily mobilizable fractions to slowly mobilizable fractions; this transformation is enhanced in the presence of crop growth and close association with the crop roots. After 18 weeks of crop growth, about 60% of Cu added as Cu(I)-S phase in the amended sludge was converted to other phases (this further decrease was associated with an increase in organo-Cu(II) phases). The proportions of Cu and Zn in the exchangeable fraction decreased over time from 36% and 70%, respectively, in the amended soil to 28% and 59% in the control experiments and to 24% and 53% with crop growth. This trend is compatible with the observation that Cu and Zn in soils tend to be in relatively unreactive phases. Current regulations governing the application of digested sewage sludge to agricultural land do not capture the changes in metal mobility during ageing and crop growth.

## Supplementary Information

Below is the link to the electronic supplementary material.

Supplementary file1 (DOC 299 KB)

## Data Availability

Data is available on request from the corresponding author.
