# Supplementary material for: Evolution of Cu and Zn speciation in agricultural soil amended by digested sludge over time and repeated crop growth
Source: Environ Sci Pollut Res Int. 2024 Aug 31;31(42):54738–52. doi: 10.1007/s11356-024-34784-8 (PMC11413148; doi:10.1007/s11356-024-34784-8)
Supplement: Supplementary file 1 — Supplementary file1 (DOC 299 KB) [file 11356_2024_34784_MOESM1_ESM.doc]

**Supplementary Information**

**Evolution of Cu and Zn speciation in agricultural soil amended by digested sludge over time and repeated crop growth**

Jianting Feng ^a, *^, Ian T. Burke ^b^, Xiaohui Chen ^a^, Douglas I. Stewart ^a^

^a^ School of Civil Engineering, University of Leeds, Leeds LS2 9JT, UK

^b^ School of Earth and Environment, University of Leeds, Leeds LS2 9JT, UK

^*^ Corresponding author: cnjf@leeds.ac.uk

**Consisting of**

9 Tables

2 Figures

3 Sections

# Section S1 Isotherm sorption of sewage sludge for Zn and Cu

To produce a sludge-amended soil where changes in Cu and Zn speciation could be easily determined by both BCR extraction and XAS techniques, the original sewage sludge obtained from Esholt wastewater treatment plant needed to be spiked with ZnCl_2_ and CuCl_2_. Therefore, we first investigated the sorption capacity of sewage sludge for Cu and Zn. Analytical grade ZnCl_2_ and CuCl_2_·2H_2_O were added to the original sewage sludge (dry solids content: 45.6 g/L) at six different ratios (0.4%, 0.8%, 1%, 2%, 5% and 8%; *m*_metals_ / *m*_dry sludge solids_). The amended sludge was shaken for 24 hours (INFORS HT Multitron Pro incubator shaker, 120 rpm, room temperature) and then centrifuged at 3000 g for 20 min, before aqueous phase was decanted off and the solids were air-dried, disaggregated, and sieved. The total Zn and Cu concentrations and their chemical forms in the amended sludge were determined by aqua regia digestion and BCR extractions. These results served as the basis for selecting the appropriate amended sludge for producing the target sludge-amended soil.

## Section S1.1 Sorption capacity of sewage sludge

When Cu and Zn were added to the original sludge to a final concentration < 2%, averagely, more than 90% of Cu and 85% of Zn were absorbed to the sludge (see Table S1). With further increases in the amount of metals being added, an increasing proportion remained in the aqueous phase. When 8% Cu and Zn were added to the sludge, less Zn was sorbed than when 5% were added, suggesting that Cu can out compete Zn for some sorption sites. According to the sorption pattern of the sludge to Cu and Zn, in combination with the reasonable application ratio of the sludge to agriculture and the target metal concentration in the sludge-amended soil of this study, the amended sludge produced by adding metals to the original sludge at a ratio of 2% is adopted for producing the target sludge-amended soil.

**Table S1 Sorption capacity of sewage sludge to Cu and Zn**

| *m*_metals_ / *m*_dry sludge solids_  ratio (%) | Sludge volume  (mL) | Dry sludge solids content (g/L) | Added metal amount to sludge  (mg) | | Absorbed metal amount to sludge  (mg) | | | Sorption ratio  (%) | |
| --- | --- | --- | --- | --- | --- | --- | --- | --- | --- |
|  |  |  | Cu | Zn | | Cu | Zn | Cu | Zn |
| 0.4 | 200 | 45.6 | 36.48 | 36.48 | | 34.54 | 32.11 | 95 | 88 |
| 0.8 | 200 | 45.6 | 72.96 | 72.96 | | 68.18 | 67.42 | 93 | 92 |
| 1 | 200 | 45.6 | 91.20 | 91.20 | | 92.68 | 76.32 | 101 | 84 |
| 2 | 200 | 45.6 | 182.40 | 182.40 | | 166.14 | 155.72 | 91 | 85 |
| 5 | 200 | 45.6 | 456.00 | 456.00 | | 368.70 | 258.09 | 81 | 57 |
| 8 | 200 | 45.6 | 729.60 | 729.60 | | 571.42 | 195.54 | 78 | 27 |

## Section S1.2 Metal speciation in amended sludges

BCR extractions indicated that Cu was principally distributed in the H_2_O_2_ extract (78%) in the original sludge and its speciation pattern was H_2_O_2_ extract > aqua regia extract > NH_2_OH·HCl extract > CH_3_COOH extract (see Fig. S1; concentration values of each Cu fraction shown in Table S2). The similar distribution pattern of Cu in the amended sludges was also observed when the added ratio of Cu was within 1%. The proportion of H_2_O_2_ extract-Cu in these amended sludges accounted for ~70%. A ~10% reduction in the proportion of the H_2_O_2_ extract-Cu and a 10% increase in the proportion of the CH_3_COOH extract-Cu were observed after adding Cu to the original sludge at a ratio of 2%. The Cu speciation pattern in this amended sludge was H_2_O_2_ extract (60%) > aqua regia extract (14%) ≈ CH_3_COOH extract (14%) ≈ NH_2_OH·HCl extract (12%). With further increasing Cu content, a significant increase in the proportion of CH_3_COOH extract-Cu occurred that reached to 42% and 61% at the ratio of 5% and 8%, respectively. Concurrently, a dramatic reduction occurred in the proportion of H_2_O_2_ extract-Cu that reduced to 35% and 25%, respectively. Also, a slight reduction appeared in the proportion of aqua regia extract-Cu. As a result, the Cu speciation in these two amended sludges was CH_3_COOH extract > H_2_O_2_ extract > NH_2_OH·HCl extract > aqua regia extract.

BCR extractions (see Fig. S1; concentration values of each Zn fraction in Table S3) also showed that Zn in the original sludge was distributed in between CH_3_COOH extract (12%), NH_2_OH·HCl extract (31%), H_2_O_2_ extract (37%), and aqua regia extract (20%). With an initial addition of Zn at a ratio of 0.4%, there was an immediately dramatic increase in the proportion of CH_3_COOH extract-Zn (increased to 61%) and an apparent reduction in the proportions of H_2_O_2_ extract (reduced to 8%) and aqua regia extract (reduced to 5%). The CH_3_COOH extract became the primary speciation of Zn in this amended sludge. A continuous increase in the proportion of CH_3_COOH extract-Zn occurred with the increasing ratios. The proportion of CH_3_COOH extract-Zn finally reached to over 90% when the added ratio was 5%.

**Fig. S1** a) Cu and b) Zn speciation in original sewage sludge and amended sludges. AS0: original sewage sludge; AS1-AS6: the sludge spiked with metals at a ratio of 0.4%, 0.8%, 1%, 2% (used for producing the amended soil), 5% and 8%, respectively

**Table S2** Cu concentration of each fraction in original and amended sludges (mg/kg of dry sludge)

| Sludges | Cu concentration in each fraction  (mg/kg) | | | | Sum of four fractions (mg/kg) | Total Cu content by aqua regia (mg/kg) | Recovery rate (%) |
| --- | --- | --- | --- | --- | --- | --- | --- |
|  | CH_3_COOH extract | NH_2_OH·HCl extract | H_2_O_2_ extract | Aqua regia extract |  |  |  |
| AS0 | 6  ± 1 | 11  ± 3 | 242  ± 2 | 50  ± 6 | 309  ± 11 | 303  ± 2 | 102 |
| AS1 | 86  ± 4 | 395  ± 12 | 2935 ± 28 | 581  ± 45 | 3997  ± 28 | 4090  ± 16 | 98 |
| AS2 | 192  ± 4 | 541  ± 6 | 5629 ± 93 | 1131  ± 30 | 7493  ± 61 | 7778  ± 18 | 96 |
| AS3 | 480  ± 25 | 871  ± 3 | 7788 ± 32 | 1844  ± 123 | 10983  ± 94 | 10465  ± 53 | 105 |
| AS4 | 2724  ± 52 | 2398  ± 72 | 11699 ± 377 | 2690  ± 464 | 19511  ± 173 | 18520  ± 202 | 105 |
| AS5 | 17702  ± 255 | 6082  ± 143 | 14723 ± 494 | 3390  ± 633 | 41896  ± 1039. | 40731  ± 176 | 103 |
| AS6 | 37597  ± 371 | 7227  ± 150 | 15685 ± 38 | 1101  ± 15 | 61609  ± 503 | 62959  ± 490 | 98 |

Values are expressed as mean ± standard deviation (*n*=3). AS0: original sewage sludge; AS1-AS6: the sludge spiked with metals at a ratio of 0.4%, 0.8%, 1%, 2%, 5% and 8%, respectively

**Table S3** Zn concentration of each fraction in original and amended sludges (mg/kg of dry sludge)

| Sludges | Zn concentration in each fraction  (mg/kg) | | | | Sum of four fractions (mg/kg) | Total Zn content by aqua regia (mg/kg) | Recovery rate (%) |
| --- | --- | --- | --- | --- | --- | --- | --- |
|  | CH_3_COOH extract | NH_2_OH·HCl extract | H_2_O_2_ extract | Aqua regia extract |  |  |  |
| AS0 | 76  ± 0 | 195  ± 3 | 231  ± 2 | 125  ± 1 | 628  ± 2 | 620  ± 7 | 101 |
| AS1 | 2483  ± 35 | 1091  ± 31 | 315  ± 9 | 190  ± 2 | 4079  ± 21 | 4140  ± 30 | 99 |
| AS2 | 5584  ± 23 | 1769  ± 17 | 353  ± 23 | 203  ± 6 | 7909  ± 40 | 8012  ± 56 | 99 |
| AS3 | 6121  ± 291 | 2039  ± 115 | 392  ± 43 | 323  ± 72 | 8875  ± 63 | 8988  ± 89 | 99 |
| AS4 | 14291  ± 272 | 2676  ± 159 | 354  ± 72 | 366  ± 71 | 17687  ± 477 | 17694  ± 129 | 100 |
| AS5 | 25730  ± 291 | 2464  ± 193 | 225  ± 21 | 295  ± 70 | 28714  ± 285 | 28919  ± 280 | 99 |
| AS6 | 19537  ± 185 | 1367  ± 90 | 181  ± 8 | 84  ± 4 | 21169  ± 267 | 22061  ± 270 | 96 |

Values are expressed as mean ± standard deviation (*n*=3). AS0: original sewage sludge; AS1-AS6: the sludge spiked with metals at a ratio of 0.4%, 0.8%, 1%, 2%, 5% and 8%, respectively

# Section S2 Quality control for aqua regia digestion

To ensure comparability with other laboratories, total Zn and Cu concentrations in the certified reference material (LSKD-2, typical lake sediments from various locations within the Canadian Shield, provided by CCRMP, CANMET Mining and Mineral Sciences Laboratories) were determined by the aqua regia digestion procedure used in this study. The digestion results show good agreement with the provisional reference values (see Table S4).

**Table S4** Comparison between total Zn and Cu concentrations in a certified reference material obtained by aqua regia digestion and provisional values

| Metal | Values of total metal content using aqua regia digestion (mg/kg) | Provisional values (mg/kg) | Recovery ratio  (%) |
| --- | --- | --- | --- |
| Cu | 36 ± 0 | 37 | 97 |
| Zn | 208 ± 5 | 209 | 100 |

Values are expressed as mean ± standard deviation (*n*=3)

# Section S3 XAS analysis

XAS spectra were collected at Cu and Zn K-edges (8979 and 9659 eV respectively) on beamline I18 at the Diamond Light Source operating at 3 GeV with a typical current of 200 mA, using a nitrogen cooled Si(111) double crystal monochromator and focussing optics. A pair of plane mirrors was used to reduce the harmonic content of the beam and Kirkpatrick-Baez mirrors were used to produce either a focused or unfocused beam (0.005 mm or 0.1 mm diameter respectively at the sample) for microfocus or bulk sample analysis as required. For standards prepared as pressed pellets, K-edge spectra were collected in transmission mode using ionisation chamber detectors. For samples and solutions, data were collected in fluorescence mode using a 4 element 1mm thick sensor Vortex Si Drifts detector with cube pre-amps. All data collection was performed at room temperature (~295 °K) within a He-filled bag (to lower the attentuation of low energy fluoressence X-rays). For the resin emedded blocks, approximately 0.5 × 0.5 mm multielment microfocus XRF spectra were collected. These where then processed by the beamline softeware in real time to produce elemental maps (for Al, Si, S, Ca, Cr, Fe, Cu, Zn and As). Only single XANES spectra was collected (~10 min) from any one spot within samples and the sample stage automatically moved to expose an unaffected part of the sample before subsequent scans. For bulk analysis, multiple scans were then averaged to improve the signal to noise ratio using Athena version 0.9.26 . For all XANES spectra, absorption was normalised in Athena over the full data range and plotted from approximately -30 eV to +30 eV relative to the edge position. Spectra were corrected for any drift in E_0_ using the data collected from the metal foil standards. Linear combination fitting (LCF) was preformed in Athena using the full range of available standards to determine the most likely combinations of standards to best fit the sample. In the LCF analysis the number of standards used was limited to a maximum of 3 to reduce the degree of freedom present and all fits were forced by the software to produce results that summed to 100%. LCF typically produced results of elemental speciation with an uncertainity of ± 4%.

The sources or synthesis of all reference standards are listed in Table S5 and S6. All reference standards XAS spectra used in the LCF are shown in Fig. S2. All the reference standards spectra used were collected on Beamline I18 at Diamond light Source. Some of Zn-XANES spectra used were shared by the authors (Prof. Fred Mosselmans, Diamond Light Source; Prof Bryne Nygwena, University of Ediburgh; [Adele et al. (2018)](#_ENREF_1)); Zn(0)-foil data collected at the same time was also provided to ensure the correct calibration of standard spectra collected in different I18 sessions.

**Fig. S2** XANES spectra of reference standards of Cu and Zn

**Table S5** Reference standards label, chemical formula, and source of reference compounds used for linear combination fitting analyses of Cu XANES data

| Reference standards sample (label/chemical formula) | Sources and/or synthesis method |
| --- | --- |
| Cu(II)CO_3_ | Purchased laboratory chemical |
| Cu(II)SO_4_ | Purchased laboratory chemical |
| Cu(II)O | Purchased laboratory chemical |
| Cu(I)_2_O | Purchased laboratory chemical |
| Covellite (Cu(I)S) | Natural mineral sample |
| Cu(II)-humic complex (Cu-HA) | 30 mL of 500 mg/kg Cu^2+^ (pH=3.28) and 3 g humic acid (Merck, UK) reacted for 24h, collected by centrifuging and dried at 40℃ |
| Cu^2+^_(aqueous)_ | 1000 mg/L Cu(NO_3_)_2_ solution |
| Cu(II)(CH_3_COO)_2_ | Purchased laboratory chemical |
| Cu(I)S nano particles (NP CuS) | Following the method of [Adele et al. (2018)](#_ENREF_1), 50 mL of 100 mM Cu^2+^ and 50 mL of 500 mM S^2-^ was reacted for 24h; separated solids recovered by centrifugation and dried at 20 ℃ in a 95%N_2_/5%H_2_ atmosphere |
| Cu(II)_3_(PO_4_)_2_ | Purchased laboratory chemical |
| Cu(II)(OH)_2_ | 10 ml of 6000 ppm Cu_2_(NO_3_)_2_ solution and 10 ml of 1M KOH reacted and collected by filtration |
| Cu(II)-hydrous ferric oxide  (Cu-HFO) | Hydrous ferric oxide synthesised according to the method of [Cornell and Schwertmann (2003)](#_ENREF_2) (containing haematite, goethite and ferrihydrite by XRD) was reacted with Cu^2+^ for 24 hours at pH 7-8. Solids recovered by filtration and dried at 20℃ |

**Table S6** Reference standards label, chemical formula, and source of reference compounds used for linear combination fitting analyses of Zn XANES data

| Reference standards sample (label/chemical formula) | Sources and/or synthesis method |
| --- | --- |
| Zn(II)SO_4_ | Spectra shared by authors - [Adele et al. (2018)](#_ENREF_1) |
| Zn(II)O | Spectra shared by authors - [Adele et al. (2018)](#_ENREF_1) |
| Sphalerite (Zn(II)S) | Natural mineral sample |
| Zn(II)-FeOOH | Geothite synthesised according to the method of [Cornell and Schwertmann (2003)](#_ENREF_2) was reacted with Zn^2+^ for 24 hours at pH 7-8. Solids recovered by filtration and dried at 40℃ |
| Zn(II)S nano particles (NP ZnS) | Spectra shared by authors - [Adele et al. (2018)](#_ENREF_1) |
| Zn(II)CO_3_ | Spectra shared by authors - [Adele et al. (2018)](#_ENREF_1) |
| Zn(II)(CH_3_COO)_2_ | Spectra shared by authors - [Adele et al. (2018)](#_ENREF_1) |
| Zn(II)_3_(PO_4_)_2_ | Spectra shared by authors - [Adele et al. (2018)](#_ENREF_1) |
| Zn^2+^ _(aqueous)_ | 1000 mg/L ZnCl_2_ solution |
| Zn(II)-humic complex (Zn-HA) | 30 mL 500 mg/kg Zn^2+^ (pH=3.28) and 3 g humic acid (Merck, UK) reacted for 24h, collected by centrifuging and dried at 40℃ |
| Zn(II)-hydrous ferric oxide (Zn-HFO) | Hydrous ferric oxide synthesised according to the method of [Cornell and Schwertmann (2003)](#_ENREF_2) (containing haematite, goethite and ferrihydrite by XRD) was reacted with Zn^2+^ for 24 hours at pH 7-8. Solids recovered by filtration and dried at 20℃ |
| Zn(II)(OH)_2_ | Precipitated from 6000 ppm ZnCl_2_ solution using 1 M KOH and collected by filtration and dried at 40℃ |

**Table S7** Characteristics of original sewage sludge and agricultural soil

| Parameter | Sewage sludge | Agricultural soil | Maximum permissible metal concentrations in UK soil (mg/kg) | | | | US limits of metals in sewage sludge (mg/kg) |
| --- | --- | --- | --- | --- | --- | --- | --- |
|  |  |  | pH  (5-5.5) | pH  (5.5-6) | pH  (6-7) | pH  (>7) | EPA legislation |
| pH | 7.9 | 7.5 |  |  |  |  |  |
| Organic matter (%) | 51.1 | 6.6 |  |  |  |  |  |
| Total Kjeldahl nitrogen  (mg/kg) | 75516.6 | 2908.0 |  |  |  |  |  |
| Total phosphorus (mg/kg) | 30206.6 | 697.3 |  |  |  |  |  |
| Cu (mg/kg) | 302.5 | 77.6 | 80 | 100 | 135 | 200 | 4300 |
| Zn (mg/kg) | 619.9 | 113.5 | 200 | 200 | 200 | 300 | 7500 |

All data are reported as mean values

**Table S8 Relative abundance of Cu species determined from LCF analysis of Cu XANES spectra of samples. LCF derived errors are given in parentheses**

| Samples | Cu species (%) | | | *R*-factor |
| --- | --- | --- | --- | --- |
|  | Cu_2_O | NP CuS | Cu-HA |  |
| Original soil | 29.9 (2.3) | 5.3 (2.4) | 64.8 (3.4) | 0.0027 |
| Original sludge | 1.9 (5) | 98.1 (5) | - | 0.0171 |
| Amended sludge | 16.1 (2.6) | 69.2 (2.7) | 14.7 (3.7) | 0.0035 |
| Amended soil | 43.2 (2.6) | 40.6 (2.7) | 16.2 (3.7) | 0.0036 |
| Control_1st round | 43.5 (2.2) | 38.4 (2.3) | 18.1 (3.2) | 0.0028 |
| Control_2nd round | 31.9 (2.2) | 29.4 (2.2) | 38.7 (3.1) | 0.0025 |
| Control_3rd round | 39.2 (2.4) | 32.0 (2.5) | 28.8 (3.4) | 0.0032 |
| Bulk_1st round | 41.9 (2.8) | 35.0 (2.9) | 23.1 (4) | 0.0041 |
| Bulk_2nd round | 40.9 (2.7) | 33.5 (2.8) | 25.6 (3.9) | 0.0038 |
| Bulk_3rd round | 40.9 (1.9) | 28.3 (2.0) | 30.7 (2.8) | 0.0020 |

**Table S9 Relative abundance of Zn species determined from LCF analysis of Zn XANES spectra of samples. LCF derived errors are given in parentheses**

| Samples | Zn species (%) | | |  | *R*-factor |
| --- | --- | --- | --- | --- | --- |
|  | NP ZnS | Zn-HFO | Zn^2+^ (aq) | |  |
| Original soil | - | 72.7 (3) | 27.3 (3) | | 0.0104 |
| Original sludge | 79.4 (1.1) | 20.6 (1.1) | - | | 0.0020 |
| Amended sludge | 22.3 (1.4) | 77.7 (1.4) | - | | 0.0032 |
| Amended soil | - | 67.9 (2.4) | 32.1 (2.1) | | 0.0045 |
| Control_1st round | - | 74.5 (2.0) | 25.5 (1.4) | | 0.0022 |
| Control_2nd round | - | 75.3 (2.1) | 24.7 (1.5) | | 0.0023 |
| Control_3rd round | - | 76.9 (2.0) | 23.1 (1.4) | | 0.0020 |
| Bulk_1st round | - | 74.8 (2.0) | 25.2 (1.4) | | 0.0021 |
| Bulk_2nd round | - | 75.6 (2.0) | 24.4 (1.4) | | 0.0020 |
| Bulk_3rd round | - | 77.3 (2.4) | 22.7 (1.9) | | 0.0040 |

**References**
